# Glycerolipid Composition of the Red Macroalga *Agarophyton Chilensis* and Comparison to the Closely Related *Agarophyton Vermiculophyllum* Producing Different Types of Eicosanoids †

**DOI:** 10.3390/md17020096

**Published:** 2019-02-02

**Authors:** Masaki Honda, Takashi Ishimaru, Yutaka Itabashi, Mikhail Vyssotski

**Affiliations:** 1 Faculty of Science & Technology, Meijo University, Shiogamaguchi, Tempaku-ku, Nagoya 468-8502, Japan; 2 Faculty of Fisheries Sciences, Hokkaido University, Minato-cho, Hakodate 041-0811, Japan; ishimaru_takashi@kaken.co.jp; 3 National Research Institute of Fisheries Science, Japan Fisheries Research and Education Agency, Yokohama 236-8648, Japan; 4 Callaghan Innovation, 69 Gracefield Road, P.O. Box 31310, Lower Hutt 5040, New Zealand; mikhail.vyssotski@callaghaninnovation.govt.nz

**Keywords:** *Agarophyton chilensis*, *Agarophyton vermiculophyllum*, lipid class, fatty acid, glycerolipid molecular species, arachidonic acid cascade

## Abstract

The red macroalga *Agarophyton chilensis* is a well-known producer of eicosanoids such as hydroxyeicosatetraenoic acids, but the alga produces almost no prostaglandins, unlike the closely related *A. vermiculophyllum*. This indicates that the related two algae would have different enzyme systems or substrate composition. To carry out more in-depth discussions on the metabolic pathway of eicosanoids between the two algae, we investigated the characteristics of glycerolipids, which are the substrates of eicosanoids production, of *A. chilensis* and compared them to the reported values of *A. vermiculophyllum*. In *A. chilensis*, monogalactosyldiacylglycerol (MGDG), digalactosyldiacylglycerol (DGDG), sulfoquinovosyldiacylglycerol (SQDG), and phosphatidylcholine (PC) were the major lipid classes and accounted for 44.4% of the total lipid extract. The predominant fatty acids were arachidonic acid (20:4n-6), an eicosanoids precursor, and palmitic acid (16:0). The 20:4n-6 content was extremely high in MGDG and PC (>70%), and the 16:0 content was extremely high in DGDG and SQDG (>40%). A chiral-phase HPLC analysis showed that fatty acids were esterified at the *sn*-1 and *sn*-2 positions of those lipids. The glycerolipid molecular species were determined by reversed-phase HPLC–ESI–MS analysis. The main glycerolipid molecular species were 20:4n-6/20:4n-6 (*sn*-1/*sn*-2) for MGDG (63.8%) and PC (48.2%), 20:4n-6/16:0 for DGDG (71.1%) and SQDG (29.4%). These lipid characteristics of *A. chilensis* were almost the same as those of *A. vermiculophyllum*. Hence, the differences of the eicosanoids producing ability between the two algae would not be due to the difference of substrate composition but the difference of enzyme system.

## 1. Introduction

**Figure 1:**
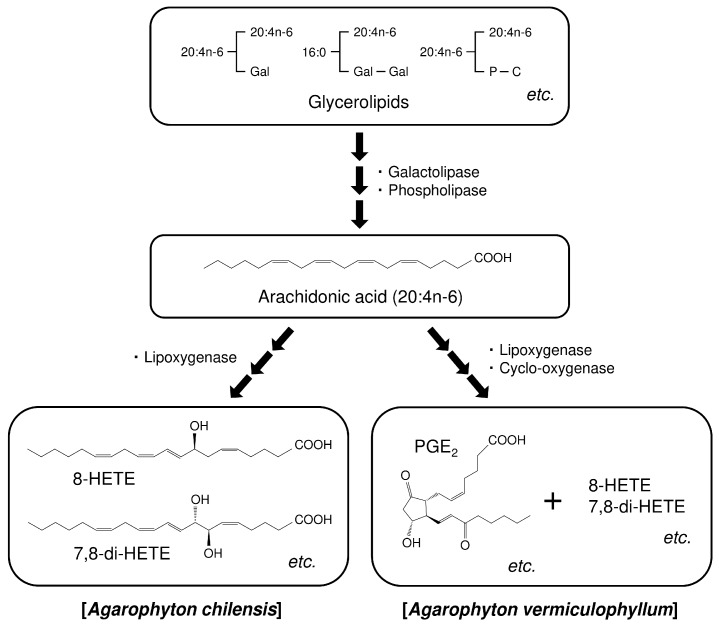
Prospective schematic diagram of arachidonic acid cascade of *Agarophyton chilensis* and *Agarophyton vermiculophyllum* [4,6,7,8,9,10,11,12,13].

Red algae are rich in polyunsaturated fatty acids (PUFA) such as arachidonic acid (20:4n-6) and eicosapentaenoic acid (20:5n-3), which are precursors of eicosanoids. The red macroalga *Agarophyton chilensis* (C.J.Bird, McLachlan et E.C.Oliveira) Gurgel, J.N.Norris et Fredericq (= *Gracilaria chilensis*) [1], which is native along Southern Hemispheric coastal regions including Chile and New Zealand, is an important marine resource in the production of agar [2,3,4]. Since the alga can be easily planted and harvested, this crop is exploited by the local population widely along the Chilean coast [4]. *A. chilensis* is a well-known producer of hydroxylated and dihydroxylated fatty acids derived from 20:4n-6, such as 8-hydroxyeicosatetraenoic acid (8-HETE) and 7,8-dihydroxyeicosatetraenoic acid (7,8-diHETE) for chemical defense against epiphytes [4,5]. On the other hand, the closely related *A. vermiculophyllum* (Ohmi) Gurgel, J.N.Norris et Fredericq (= *G. vermiculophylla*) [1] produces prostaglandins from 20:4n-6, such as PGE_2_ and PGF_2α_, in addition to 8-HETE and 7,8-diHETE [4,6,7,8,9,10] (Figure 1), which indicates that those algae would have different enzyme systems or substrate composition in spite of related species. Algal eicosanoids are produced from glycerolipid substrates glyceroglycolipids and phospholipids (PC), which are hydrolyzed by acyl-hydrolases that are activated by physical wounding [4,7,11,12]. The wounding releases 20:4n-6 or 20:5n-3, which are then transformed to eicosanoids by various enzymes (e.g., lipoxygenases, which are deeply relevant to 8-HETE and 7,8-diHETE production [4,5]; and cyclooxygenases, which are deeply relevant to prostaglandins production [12,13]). Ample studies have investigated the arachidonate metabolites and found their different compositions among Gracilariales (Rhodophyta) to date [4,5,6,7,8], but few studies have focused on the details of the substrates glyceroglycolipids and phospholipids. Therefore, to discuss the differences of the arachidonic acid cascade among Gracilariales, a critical analysis of the substrates is important. Very recently, we reported lipid characteristics of *A. vermiculophyllum* [14]. In the present study, to carry out more in-depth discussions on the metabolic pathway of eicosanoids among Gracilariales, lipid characteristics of *A. chilensis* were investigate and compared with those of *A. vermiculophyllum* [14], which biosynthesizes the different arachidonate metabolites [4]. Namely, lipid classes, fatty acid composition, and glycerolipid molecular species of *A. chilensis* were clarified using gas chromatography (GC), high-performance liquid chromatography (HPLC), and mass spectrometry (MS), and compared with those of *A. vermiculophyllum* obtained from our previous study [14].

## 2. Results and Discussion

### 2.1. Lipid Class Composition

**Figure 2:**
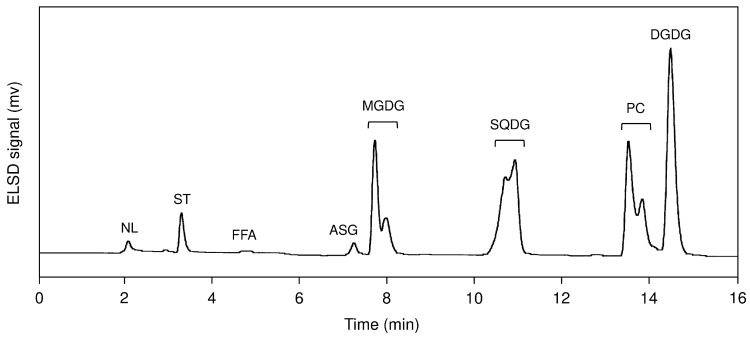
HPLC–ELSD chromatogram of total lipids from *Agarophyton chilensis*. Peaks were identified by comparison of retention times with those of standard lipids. NL, neutral lipid; ST, sterol; FFA, free fatty acid; ASG, acyl steryl glycoside.

The HPLC–evaporative light–scattering detection (ELSD) analysis clearly showed that *A. chilensis* contained three glyceroglycolipids—monogalactosyldiacylglycerol (MGDG), digalactosyldiacylglycerol (DGDG), and sulfoquinovosyldiacylglycerol (SQDG)—and a phospholipid (PC), as well as some lipids such as acyl steryl glycosides (ASG) and sterols (ST) (Figure 2). Those glycerolipids were also found in other Gracilariale (e.g., *A. vermiculophyllum*, *G. textorii*, and *G. gigas* [14,15,16]). Da Costa et al. [17]. reported that some minor phospholipids such as phosphatidylinositol (PI) and phosphatidylethanolamine (PE) were detected from Gracilariales using MS analysis. Those phospholipids could not be detected in this study, probably because of the dynamic range and limited sensitivity of the ELSD detector [18]. Quantitative estimations of the glycerolipids (MGDG, DGDG, SQGD, and PC) were carried out by HPLC–ELSD using a calibration curve for each glycerolipid [14]. The sum of the four glycerolipids of *A. chilensis* accounted for 44.4% of the total lipid extract, the contents of which (mg/g of total lipids ± SD of three analyses) were higher in the order of DGDG (142.2 ± 2.9 mg/g) > SQDG (130.8 ± 6.1 mg/g) > PC (104.5 ± 3.6 mg/g) > MGDG (66.5 ± 2.7 mg/g) (Table 1). A similar order had been observed in *A. vermiculophyllum* in our previous report [14]: DGDG (123.7 ± 2.9 mg/g) > PC (110.7 ± 8.9 mg/g) ≈ SQDG (107.8 ± 5.8 mg/g) > MGDG (88.1 ± 1.2 mg/g). On the other hand, the composition ratio can change in some degree depending on geographical factors and the season [19]. Some other Gracilariales had different glycerolipid compositions (e.g., in *G. bursa-pastoris* and *G. chorda*, MGDG was the most predominant glycerolipid, and in *G. textorii* and *G. gigas*, PC was the richest one [15]). The results obtained in this study indicated that MGDG, DGDG, SQGD, and PC would become the main substrates of eicosanoids production in *A. chilensis* as well as *A. vermiculophyllum* [14].

### 2.2. Fatty Acid Composition of Glycerolipids

Table 2 shows the fatty acid compositions of the glycerolipids from *A. chilensis* examined in this study and *A. vermiculophyllum* obtained from our previous report [14]. The main fatty acids of the total lipids in *A. chilensis* were 16:0 (27.4%) and 20:4n-6 (58.9%), as well as in *A. vermiculophyllum* (Table 2) [14]. Also, in other Gracilariales such as *A. tenuistipitatum* (= *G. tenuistipitata*), *G. debilis*, *G. dura*, and *G. textorii*, 16:0 and 20:4n-6 were the major fatty acids [15,20,21]. On the other hand, the most predominant fatty acid in *G. changgi* and *G. gigas* was 20:5n-3 [15,22]; *G. folifera* and *Hydropuntia edulis* (= *G. edulis*) contained almost no PUFAs [23]. The characteristics of fatty acid composition depended on each major glycerolipid (MGDG, DGDG, SQDG, and PC) of *A. chilensis*. The 20:4n-6 content was especially high (>60%) in MGDG and PC, and the 16:0 content was especially high (>50%) in DGDG and SQDG. PC contained a large amount of PUFAs (76.6%), whereas SQDG was rich in saturated fatty acids (65.4%). A similar composition was also observed in *A. vermiculophyllum* [14] and *G. chorda* [15]. These results suggest that MGDG and PC rich in 20:4n-6 would become the main substrates of eicosanoids production in *A. chilensis* and *A. vermiculophyllum*.

### 2.3. Positional Isomers of Glycerolipids

Generally, in higher plants such as spinach and Arabidopsis, the glycerol moieties of glycerolipid have the *S* configuration [24], as well as in algae such as *A. vermiculophyllum* [14] and *Sargassum yezoense* [25]. In this study, the binding position of fatty acids with the glycerol moieties was determined using chiral-phase HPLC. The chiral-phase HPLC chromatograms of 3,5-dinitrophenylurethane (3,5-DNPU) derivatives prepared from the *sn*-1,2(2,3)-diacylglycerols (DAG) standard derived from tuna orbital oil triacylglycerols (TAG) and the DAG released from MGDG, DGDG, SQDG, and PC of *A. chilensis* are shown in Supplementary Figure S1. The standard was clearly separated into two groups, representing the *sn*-1,2- and *sn*-2,3-enantiomers (Supplementary Figure S1A): The faster elution group consisted of *sn*-1,2-enantiomers, and the subsequent group consisted of *sn*-2,3-enantiomers on the A-K03 column [14,25,26,27]. Since the presence of various molecular species in tuna orbital oil TAG, each enantiomeric group was split. All DAG released from MGDG, DGDG, SQDG, and PC of *A. chilensis* were eluted within 20 min, and the enantiomeric group was also split because of the presence of various molecular species (Supplementary Figure S1B–E). Thus, in *A. chilensis*, fatty acids were esterified at the *sn*-1 and *sn*-2 positions (*S* configuration) of those glycerolipids as with higher plants [24] and *A. vermiculophyllum* [14].

### 2.4. Molecular Species of Glycerolipids

**Figure 3:**
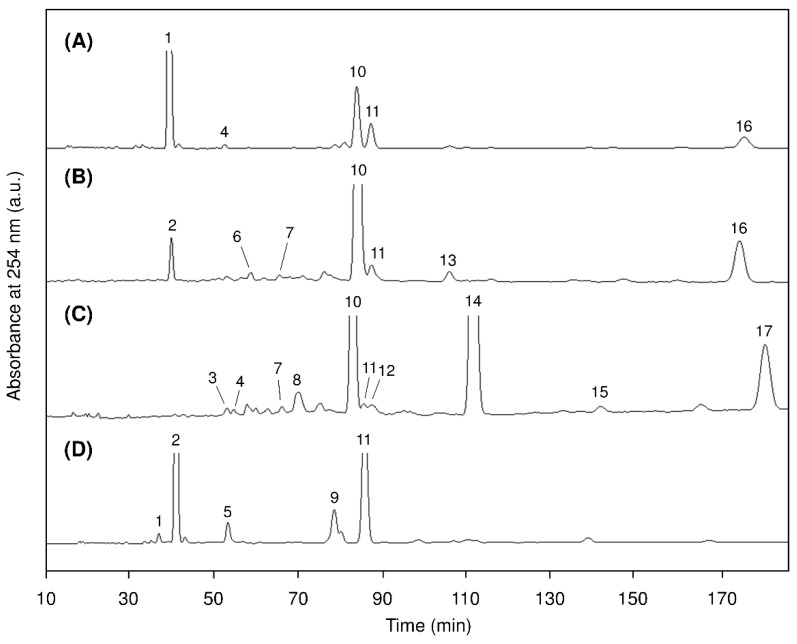
Reversed-phase HPLC chromatograms of the 3,5-DNPU derivatives of diacylglycerols released from (**A**) MGDG, (**B**) DGDG, (**C**) SQDG, and (**D**) PC of *Agarophyton chilensis*. Peak numbers corresponding to those in Table 3.

Figure 3 shows the reversed-phase HPLC chromatograms of 3.5-DNPU derivatives and Table 3 shows the molecular species composition of the glycerolipids from *A. chilensis* examined in this study and *A. vermiculophyllum* obtained from our previous report [14]. 

The quantitative estimation of the glycerolipid molecular species were performed by reversed-phase HPLC with UV detection, and the detected molecular species were determined by reversed-phase HPLC–electrospray ionization (ESI)–MS analysis [14,25]. The 3,5-DNPU derivatives of the DAG released from MGDG, DGDG, SQDG, and PC were eluted in the order of the equivalent carbon number (ECN; total number of carbon atoms in the two constituent fatty acids – 2 × total number of their double bonds) on the reversed-phase HPLC as previously described [14]. Although *A. vermiculophyllum* contained more kinds of the molecular species than *A. chilensis* in any glycerolipids, the major molecular species were almost the same between them. Namely, in *A. chilensis*, the 20:4n-6/20:4n-6 (*sn*-1/*sn*-2) species accounted for the largest proportion of MGDG (63.8%) and PC (48.2%), whereas the 20:4n-6/16:0 species was the predominant species in DGDG (71.1%), and the 14:0/16:0 and 20:4n-6/16:0 species mainly presented in SQDG (49.3% and 29.4%, respectively). A similar molecular species composition was observed in *A. vermiculophyllum* [14]—56.5% and 40.0% of the 20:4n-6/20:4n-6 species in MGDG and PC, respectively; 75.4% of the 20:4n-6/16:0 species in DGDG; 49.3% of the 14:0/16:0 and 29.4% of the 20:4n-6/16:0 in SQGD. MGDG and PC of *A. chilensis* also contained a high proportion of 20:4n-6/16:0 (16.2%) and 16:0/20:4n-6 (31.3%), respectively, as with *A. vermiculophyllum* [14]. The *sn*-position of 16:0 and 20:4n-6 in MGDG, DGDG, and SQDG was opposite to that of the main molecular species of PC. The 16:0 and 20:4n-6 were mainly esterified at the *sn*-1 and *sn*-2 positions, respectively, in MGDG, DGDG, and SQDG, while 16:0 and 20:4n-6 were esterified at the *sn*-2 and *sn*-1 positions, respectively, in PC. Furthermore, in addition to the 20:4n-6/16:0 species, a small amount of its reverse isomer (16:0/20:4n-6) was present in MGDG, DGDG, and SQDG, whereas PC did not contain the reverse isomer of the 16:0/20:4n-6 species. Such a tendency has also been observed in *A. vermiculophyllum* [14]. The 16:0/20:4n-6 was found in MGDG of two species of red algae, *Porphyra yezoensis* and *Corallina pilulifera*, but the species was not found in DGDG and SQDG, nor was the 20:4n-6/16:0 found in the PC of these algal species [28]. The co-existence of both isomers indicates that glycerolipids of *A. chilensis* were biosynthesized from both the prokaryotic pathway (biosynthesized in the chloroplast envelope) and the eukaryotic pathway (biosynthesized in the chloroplast envelope after passing through the endoplasmic reticulum) as described previously [14,29,30,31].

Lipid composition and glycerolipid molecular species between *A. chilensis* and *A. vermiculophyllum* were almost the same. These results indicate that the differences of the eicosanoid producing ability do not result from the difference in substrates but from the difference in enzyme systems inherent in the alga body, whereas it cannot be denied that the difference is caused by geographical factors and seasons [8,19]. Therefore, in *A. chilensis* and *A. vermiculophyllum*, the first stage reaction of eicosanoids production of the both algae is the hydrolysis of membrane glycerolipids by acyl-hydrolases, which is triggered by physical wounding [4,5], and the 20:4n-6, an eicosanoid precursor, would mainly be released from the MGDG (20:4n-6/20:4n-6 and 20:4n-6/16:0 species), DGDG (20:4n-6/16:0 species), SQDG (20:4n-6/16:0 species), and PC (20:4n-6/20:4n-6 and 16:0/20:4n-6 species).

## 3. Materials and Methods

### 3.1. Materials

The sample of *A. chilensis* was collected from the coast of Petone, Wellington, New Zealand, in May 2008. The alga was freed from epiphytic organisms and kept at −20 °C until use. Analytical- and HPLC-grade solvents, and 1,1-dimethylhydrazine were obtained from Kanto Chemical (Tokyo, Japan). Standard samples of MGDG, DGDG, and SQDG from plant leaves were obtained from Lipid Products (Redhill, United Kingdom), and PC from soybean was obtained from Sigma-Aldrich (St. Louis, MO, USA). 3,5-Dinitrophenyl isocyanate was purchased from Sumika Chemical Analysis Service (Osaka, Japan).

### 3.2. Lipid Extraction

The Bligh–Dyer method [32] was used to extract lipid from *A. chilensis*. In brief, 96.8 g of the algal sample was homogenized with 200 mL of methanol and 100 mL of chloroform (CH_3_Cl). After filtration, 100 mL of CH_3_Cl and 100 mL of water were added to the algal residue, homogenized, and filtered. Subsequently, 200 mL of CH_3_Cl was added to the residue, separated in the same manner, and then all the obtained solvent fractions were mixed. After being left standing for 12 h in the dark at room temperature, the organic solvent layer (lower layer) was collected, the solvent was evaporated to dryness under reduced pressure, and consequently 619 mg of lipid extract was obtained.

### 3.3. Lipid Class Analysis

The quantitative analysis of the major glycerolipids (MGDG, DGDG, SQDG, and PC) was performed by HPLC–ELSD according to the method described previously [14,33,34]. In brief, glycerolipids were separated with a LiChrospher 100 DIOL column (250 × 4.0 mm i.d., 5 μm particles, Merck, Darmstadt, Germany) using two mobile phases consisting of (A) CH_3_Cl and (B) methanol/acetone/water/acetic acid (30:60:9:1, *v*/*v*/*v*/*v*). The gradient profile was as follows: 0−1 min, 0% (B); 1–2 min, 0–30% (B) linear; 2–6 min, 30% (B); 6–8 min, 30–50% (B) linear; 8–13 min, 50% (B); 13–15 min, 50–100% (B) linear; 15−16 min, 100% (B). The other analytical conditions were as following: Flow rate of 0.9 mL/min; column temperature of 35 °C; detector (SEDEX model 55; SEDERE, Alfortville, France) temperature of 50 °C; nebulizer pressure (air) of 250 kPa. Peaks were identified by comparing retention times with those of standard lipids, and standard curves were prepared for MGDG, DGDG, SQDG, and PC quantifications.

### 3.4. Isolation of Glycerolipids

The major glycerolipids (MGDG, DGDG, SQDG, and PC) were separated from the total lipids of *A. chilensis* by thin-layer chromatography (TLC) on silica gel 60 F_254_ glass sheets (20 × 20 cm, 0.25 mm thick, Merck, Darmstadt, Germany), using CH_3_Cl/methanol/water/ethyl acetate/2-propanol (5:2:1:5:5, *v*/*v*/*v*/*v*/*v*) as the developing solvent [14]. SQDG was further purified by TLC, with a solvent system of CH_3_Cl/acetone/methanol/water/acetic acid (10:6:2:1:2, *v*/*v*/*v*/*v*/*v*).

### 3.5. Fatty Acid Analysis

The fatty acids in the isolated glycerolipids (MGDG, DGDG, SQDG, and PC) were methylated to fatty acid methyl esters (FAME) by heating at 90 °C for 1 h in 5% (*w*/*v*) HCl in methanol [35]. Gas chromatography analysis of the FAME was performed using a Shimadzu GC-14A (Shimadzu, Kyoto, Japan) equipped with a flame-ionization detector and an Omegawax 320 column (30 m × 0.32 mm i.d., Supelco, Bellefonte, PA, USA) [10,14]. The column temperature was elevated from 170 to 230 °C at 1 °C/min and the other conditions were as following: injector temperature, 230 °C; detector temperature, 230 °C; carrier gas, helium (linear flow 1.2 mL/min); split ratio, 1:50. Peaks were monitored on a Shimadzu Chromatopac C-R6A integrator and identified by comparing the retention data of authentic standards and known fatty acids from marine algae [14,36].

### 3.6. Release of DAG from Glycerolipids

The isolated glycerolipids (MGDG, DGDG, SQDG, and PC) were converted into DAG as described by Heinze et al. [37]. In detail, 2 mg of glycoglycerolipid and 52 mg of HIO_4_·4H_2_O were dissolved in 1 mL of methanol and placed in the dark at room temperature for 90 min. Subsequently, 4 mL of CH_3_Cl and 2.5 mL of 0.45% NaCl solution were added and shaken vigorously. After brief centrifugation, the organic solvent layer (lower layer) was evaporated under nitrogen gas. The residue was dissolved in 0.5 mL of CH_3_Cl/water/2-propanol/acetic acid (6:7:2:3, *v*/*v*/*v*/*v*) containing 1% 1,1-dimethylhydrazine and placed in the dark at 25 °C for 4 h for DGDG, and 20 h for MGDG, SQDG and PC. Then, 3 mL of hexane was added and the mixture was washed twice with 2 mL of 50 mM KH_2_PO_4_ solution and dried over anhydrous Na_2_SO_4_. The residue containing DAG released from the glycerolipids was obtained after removal of the solvent.

### 3.7. Preparation of 3,5-Dinitrophenylurethane Derivatives

The DAG released from glycerolipids (MGDG, DGDG, SQDG, and PC) were immediately converted into their 3,5-DNPU derivatives as described previously [14,36]. By this derivatization, the DAG derivative took on good absorption at 254 nm. The reaction products (DAG) and 3,5-dinitrophenyl isocyanate (5 mg) were dissolved in dry toluene (5 mL) containing 30 μL of dry pyridine, and the solution was kept at 30 °C for 3 h with stirring. After removal of the solvent by nitrogen flow, the residue (crude urethane derivatives) were purified by TLC on silica gel 60 F_254_ glass sheets (Merck, Darmstadt, Germany) with hexane/dichloromethane (CH_2_Cl_2_)/ethanol (40:10:3, *v*/*v*/*v*) as the developing solvent. Bands were visualized under UV irradiation (Rf: 0.6–0.7), and the adsorbent containing the derivatives was scraped off and extracted with diethyl ether.

### 3.8. Chiral-Phase HPLC

Chiral-phase HPLC analysis was carried out to determine the binding position of fatty acids to the glycerol moieties of the DAG as 3,5-DNPU derivatives [14,25]. The analysis was carried out on a Shimadzu LC-6A instrument equipped with a chiral column (YMC-Pack A-K03, 250 × 4.6 mm i.d., 5 μm particles, YMC, Kyoto, Japan). The mobile phase consisted of a mixture of hexane/ CH_2_Cl_2_/ethanol (40:10:1, *v*/*v*/*v*) and the detection wavelength of the derivatives was set at 254 nm. The flow rate and column temperature were set at 1 mL/min and 10 °C. The chirality of the DAG released from glycerolipids was determined by comparing the retention times of the 3,5-DNPU derivatives with those of standard *sn*-1,2- and *sn*-2,3-DAG prepared from tuna orbital oil (TAG) by partial Grignard degradation [14,24].

### 3.9. Reversed-Phase HPLC

Molecular species analysis of DAG as 3,5-DNPU derivatives was performed by reversed-phase HPLC according to the method described previously [14,25]. Briefly, the analysis was performed on a Superspher 100 RP-18 column (250 × 4.0 mm i.d., 4 μm particles, Merck, Darmstadt, Germany) with acetonitrile at a flow rate of 0.5 mL/min and a column temperature was set at 20 °C. The quantification of the 3,5-DNPU derivatives was performed by peak area integration at 254 nm (L-7455; Hitachi Ltd., Tokyo, Japan).

### 3.10. Reversed-Phase HPLC–ESI–MS

In order to determine the molecular species of DAG as 3,5-DNPU derivatives, reversed-phase HPLC–ESI–MS was carried out in the negative ion mode with an LCQ ion-trap mass spectrometer (Thermo Separation Products, San Jose, CA, USA) [14,25]. Separations of the 3,5-DNPU derivatives were performed under the same conditions as the HPLC analysis with UV detection as mentioned above. The heated capillary temperature was 270 °C. The tube lens offset, capillary voltage, and spray voltage were −60 V, −28 V, and 4.2 kV, respectively. Flow rates of the nitrogen sheath and auxiliary gases were set to 80 and 30 arbitrary units (arb), respectively. The mass spectra were taken in a mass range of m/z 150–m/z 1200. The deprotonated molecule ion ([M−H]^–^) and carboxylate anion ([RCOO]^–^) were used to identify individual molecular species of glycerolipids. The relative intensities of two carboxylate anions—carboxylate anions generated from the *sn*-1 position (R^1^COO)^–^, and carboxylate anions from the *sn*-2 position (R^2^COO)^–^—produced from 3,5-DNPU derivatives of glycerolipids by collision-induced dissociation (CID) were used for determining the *sn*-position (*sn*-1 or *sn*-2) of the acyl groups in the molecules [14,25,38]. Namely, the property that the intensities of (R^2^COO)^–^ were higher than those of (R^1^COO)^–^ when CID energy was added was utilized.

## 4. Conclusions

Lipid class, fatty acid composition, and glycerolipid molecular species of the red alga *A. chilensis* were investigated and compared with the related *A. vermiculophyllum* to deepen discussions on the different metabolic pathway of eicosanoids between them. In *A. chilensis*, MGDG, DGDG, SQDG, and PC were the major lipid classes and 20:4n-6 and 16:0 were the predominant fatty acids in these glycerolipids. The 20:4n-6/20:4n-6 species was predominant in MGDG (63.8%) and PC (48.2%), the 20:4n-6/16:0 species was predominant in DGDG (71.1%), and the 14:0/16:0 and 20:4n-6/16:0 species were predominant in SQDG (49.3% and 29.4%, respectively). The glycerolipids characteristics of *A. chilensis* were almost the same as those of *A. vermiculophyllum*. Since glycerolipids are a substance of eicosanoids production, the differences of the eicosanoids producing ability between the algae would be due to having the different arachidonic acid cascades. The results of this study should help the elucidation of the eicosanoids production mechanism of the Gracilariales. It is expected that a future study will extend to enzymes such as lipoxygenases and cyclooxygenases and the enzyme genes associated with biosynthesis of the eicosanoids the species.

## Figures and Tables

**Table 1:** Major glycerolipid composition (mg/g of total lipids ± SD of three analyses) of the red algae *Agarophyton chilensis* and *Agarophyton vermiculophyllum* as determined by HPLC–evaporative light–scattering detection (ELSD).

Species	MGDG	DGDG	SQDG	PC	Total
*A. chilensis*	66.5 ± 2.7 ^d^	142.2 ± 2.9 ^a^	130.8 ± 6.1 ^b^	104.5 ± 3.6 ^c^	444.0
*A. vermiculophyllum* *	88.1 ± 1.2	123.7 ± 2.9	107.8 ± 5.8	110.7 ± 8.9	430.3

^a–d^ Means in the same raw with different superscripts significantly differ (Tukey’s test, *p* < 0.05). * Values are obtained from the literature [14]. MGDG, monogalactosyldiacylglycerol; SQDG, sulfoquinovosyldiacylglycerol; PC, phosphatidylcholine; DGDG, digalactosyldiacylglycerol.

**Table 2:** Fatty acid composition (wt%) of the red algae *Agarophyton chilensis* and *Agarophyton vermiculophyllum*.

Fatty Acid	*A.* *Chilensis*					*A. Vermiculophyllum* *				
	Total Lipid	Lipid Class				Total Lipid	Lipid Class			
		MGDG	DGDG	SQDG	PC		MGDG	DGDG	SQDG	PC
12:0	0.3	tr	tr	0.2	nd	0.3	0.1	0.2	1.1	tr
14:0	3.6	0.4	0.8	10.9	0.9	5.3	1.7	1.8	14.7	1.2
15:0	0.2	0.1	0.3	0.5	0.1	0.6	1.1	0.8	1.0	0.2
16:0	27.4	12.5	43.8	52.6	15.2	31.6	21.7	54.0	61.7	15.3
iso 17:0	0.2	nd	nd	0.2	nd	0.6	nd	nd	nd	nd
17:0	tr	nd	nd	nd	0.2	0.4	1.1	0.3	0.2	0.2
18:0	0.5	0.4	0.6	0.8	0.7	0.7	0.5	0.4	0.4	0.7
22:0	tr	0.1	0.2	0.2	nd	0.1	nd	nd	nd	nd
Σ Saturated	32.2	13.5	45.7	65.4	17.1	39.6	26.2	57.5	79.1	17.6
16:1n-9	0.2	tr	tr	0.2	nd	3.7	1.5	0.7	1.3	tr
16:1n-7	0.1	nd	nd	0.9	0.2	tr	nd	nd	nd	0.7
18:1n-9	4.3	3.9	6.6	0.1	2.4	3.3	3.2	3.8	0.8	2.1
18:1n-7	1.5	0.3	0.2	tr	3.0	1.7	0.5	0.2	0.3	3.2
20:1n-9	0.1	0.1	tr	nd	0.1	0.2	0.2	tr	tr	nd
24:1n-9	0.2	0.2	0.1	0.2	nd	tr	nd	nd	nd	nd
Σ Monounsaturated	6.4	4.5	6.9	1.4	5.7	8.9	5.3	4.7	2.4	6.0
16:3n-3	nd	nd	nd	nd	nd	0.2	0.9	tr	nd	nd
16:4n-3	tr	nd	nd	nd	nd	0.2	0.7	nd	nd	tr
18:2n-6	0.4	0.2	0.3	0.1	1.2	0.8	0.4	0.5	0.2	0.9
18:3n-6	0.1	tr	tr	tr	0.8	0.4	0.1	tr	tr	1.1
18:3n-3	tr	nd	nd	nd	0.3	0.3	0.4	0.2	0.1	0.1
18:4n-3	nd	nd	nd	nd	nd	0.4	1.0	0.2	nd	0.2
20:2n-6	0.2	0.1	0.2	tr	0.1	0.2	0.2	0.2	nd	0.2
20:3n-6	0.5	0.2	0.2	tr	1.0	2.0	0.8	0.6	0.3	5.5
20:4n-6	58.9	80.5	46.0	32.3	72.7	44.6	63.4	35.2	17.2	63.8
20:5n-3	0.4	0.6	0.2	tr	0.4	1.7	1.6	0.5	tr	2.3
22:4n-6	tr	nd	nd	nd	0.2	0.2	tr	nd	nd	0.5
Σ Polyunsaturated	60.5	81.6	46.9	32.4	76.7	51.0	68.1	37.4	17.8	74.6
Others	0.9	0.4	0.5	0.8	0.5	0.5	0.4	0.4	0.7	1.8

* Values are obtained from the literature [14]. tr: trace (<0.1%). nd: not detected substantially.

**Table 3:** Molecular species composition (mol%) of the red algae *Agarophyton chilensis* and *Agarophyton vermiculophyllum*.

Peak No *	ECN **	Molecular Species	*A.* *Chilensis*				*A. Vermiculophyllum* ***			
		(*sn*-1/*sn*-2)	MGDG	DGDG	SQDG	PC	MGDG	DGDG	SQDG	PC
–	18	20:5n-3/16:4n-3	nd	nd	nd	nd	2.0	nd	nd	nd
–	20	18:4n-3/18:4n-3	nd	nd	nd	nd	1.0	nd	nd	nd
–	20	20:5n-3/16:3n-3	nd	nd	nd	nd	0.8	nd	nd	nd
–	20	20:5n-3/18:4n-3	nd	nd	nd	nd	0.9	1.1	nd	nd
–	22	16:2n-6/16:3n-3	nd	nd	nd	nd	0.8	nd	nd	nd
–	22	20:4n-6/20:5n-3	nd	nd	nd	nd	1.0	nd	nd	nd
–	22	20:5n-3/16:2n-6	nd	nd	nd	nd	nd	0.5	nd	nd
–	24	14:0/18:4n-3	nd	nd	nd	nd	0.5	nd	nd	nd
–	24	16:2n-6/16:2n-6	nd	nd	nd	nd	0.8	nd	nd	nd
1	24	20:4n-6/18:3n-6	nd	nd	nd	1.0	nd	nd	nd	1.2
2	24	20:4n-6/20:4n-6	63.8	4.1	nd	48.2	56.5	7.8	0.8	40.0
–	26	20:5n-3/20:2n-6	nd	nd	nd	nd	nd	nd	nd	0.9
–	26	18:2n-6/20:4n-6	nd	nd	nd	nd	nd	0.2	nd	nd
3	26	20:4n-6/14:0	nd	nd	0.4	nd	nd	0.4	0.3	nd
4	26	14:0/20:4n-6	0.8	nd	0.2	nd	1.8	1.7	0.2	nd
5	26	20:4n-6/20:3n-6	nd	nd	nd	3.2	nd	nd	nd	10.2
6	26	20:5n-3/16:0	nd	0.8	nd	nd	nd	0.5	0.2	nd
7	27	20:4n-6/15:0	nd	0.5	0.4	nd	nd	1.0	0.7	nd
–	27	15:0/20:4n-6	nd	nd	nd	nd	nd	0.3	nd	nd
8	28	12:0/16:0	nd	nd	2.7	nd	nd	nd	3.2	nd
9	28	18:1n-9/20:4n-6	nd	nd	nd	7.6	nd	nd	nd	7.7
10	28	20:4n-6/16:0	16.2	71.1	29.4	nd	17.7	75.4	58.4	nd
11	28	16:0/20:4n-6	6.7	1.8	0.3	31.3	7.3	4.3	0.2	26.3
12	29	15:0/14:0	nd	nd	0.4	nd	nd	nd	0.7	nd
–	30	16:1n-9/16:0	nd	nd	nd	nd	nd	nd	1.2	nd
13	30	14:0/18:1n-9	nd	1.7	nd	nd	0.3	0.8	nd	nd
–	30	16:0/20:3n-6	nd	nd	nd	nd	nd	nd	nd	3.8
14	30	14:0/16:0	nd	nd	49.3	nd	nd	nd	29.4	nd
–	30	16:0/20:3n-6	nd	nd	nd	nd	0.3	0.6	nd	nd
15	31	15:0/16:0	nd	nd	0.3	nd	nd	nd	nd	nd
16	32	16:0/18:1n-9	5.6	13.4	nd	nd	2.5	3.5	nd	nd
17	32	16:0/16:0	nd	nd	11.7	nd	nd	nd	3.6	nd
	Others		6.9	6.6	4.9	8.7	5.8	1.9	1.1	9.9

* Peak numbers corresponding to those given in Figure 3. ** Equivalent carbon number (total number of carbon atoms in the two constituent fatty acids − 2 × total number of their double bonds). *** Values are obtained from the literature [14]. tr: trace (<0.1%). nd: not detected substantially.

## Supplementary Materials

The following are available online at http://www.mdpi.com/1660-3397/17/2/96/s1, Figure S1: Chiral-phase HPLC chromatograms of the 3,5-DNPU derivatives of (**A**) standard sn-1,2(2,3)-DAG generated from tuna orbital oil TAG by partial Grignard degradation, and of the diacylglycerols released from (**B**) MGDG, (**C**) DGDG, (**D**) SQDG, and (**E**) PC of Agarophyton chilensis.
