# Supplementary material for: Glycerolipid Composition of the Red Macroalga Agarophyton Chilensis and Comparison to the Closely Related Agarophyton Vermiculophyllum Producing Different Types of Eicosanoids
Source: Mar Drugs. 2019 Feb 2;17(2):96. doi: 10.3390/md17020096 (PMC6410328; doi:10.3390/md17020096)
Supplement: Supplementary file 1 [file marinedrugs-17-00096-s001.pdf]

marine drugs

Supplementary Materials

Glycerolipid Composition of the Red Macroalga *Agarophyton Chilensis* and Comparison to the Closely Related *Agarophyton Vermiculophyllum* Producing Different Types of Eicosanoids †

Masaki Honda [1,*], Takashi Ishimaru [2], Yutaka Itabashi [2,3,*] and Mikhail Vyssotski [4]

1 Faculty of Science & Technology, Meijo University, Shiogamaguchi, Tempaku-ku, Nagoya 468-8502, Japan
2 Faculty of Fisheries Sciences, Hokkaido University, Minato-cho, Hakodate 041-0811, Japan; ishimaru_takashi@kaken.co.jp
3 National Research Institute of Fisheries Science, Japan Fisheries Research and Education Agency, Yokohama 236-8648, Japan
4 Callaghan Innovation, 69 Gracefield Road, P.O. Box 31310, Lower Hutt 5040, New Zealand; mikhail.vyssotski@callaghaninnovation.govt.nz
* Correspondence: honda@meijo-u.ac.jp (M.H.); yutaka@fish.hokudai.ac.jp (Y.I.); Tel.: +81-52-838-2284 (M.H.); +81-45-788-7615 (Y.I.)
† This study was carried out in Hokkaido University, Minato-cho, Hakodate 041-0811, Japan

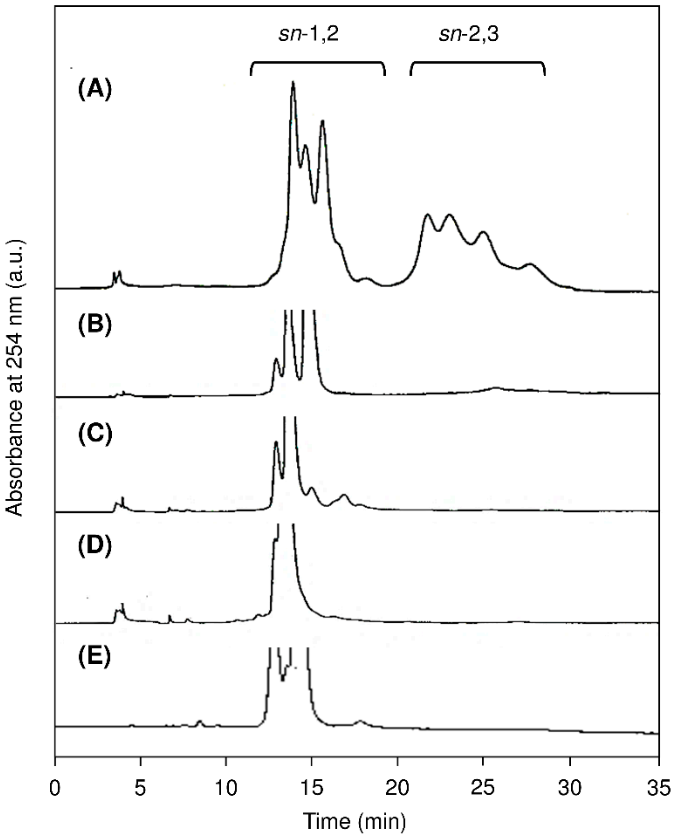

Figure S1. Chiral-phase HPLC chromatograms of the 3,5-DNPU derivatives of (**A**) standard *sn*-1,2(2,3)-DAG generated from tuna orbital oil TAG by partial Grignard degradation, and of the diacylglycerols released from (**B**) MGDG, (**C**) DGDG, (**D**) SQDG, and (**E**) PC of *Agarophyton chilensis*.
